# Lay-therapist-delivered, low-intensity, psychosocial intervention for refugees and asylum seekers (PROSPER): protocol for a pilot randomised controlled trial

**DOI:** 10.1186/s13063-020-04310-5

**Published:** 2020-04-28

**Authors:** Rebecca Rawlinson, Rabeea’h W. Aslam, Girvan Burnside, Anna Chiumento, Malena Eriksson-Lee, Amy Humphreys, Naila Khan, Daniel Lawrence, Rachel McCluskey, Annette Mackinnon, Lois Orton, Atif Rahman, Ewan Roberts, Anna Rosala-Hallas, Rhiannon Tudor Edwards, Philomene Uwamaliya, Ross G. White, Eira Winrow, Christopher Dowrick

**Affiliations:** 1 Liverpool Clinical Trials Centre, University of Liverpool, Institute in the Park, Alder Hey Children’s NHS Foundation Trust, Liverpool, L12 2AP UK; 2 PRIME Centre Wales, Health Services Research Team, Swansea University Medical School, Institute of Life Sciences 2, Floor 2, Singleton Park, Swansea, SA2 8PP UK; 3 Department of Biostatistics, University7 of Liverpool, Block F Waterhouse Building, 1-5 Brownlow Street, Liverpool, L69 3GL UK; 4 Department of Psychological Sciences, University of Liverpool, Block B Waterhouse Building, 1-5 Brownlow Street, Liverpool, L69 3GL UK; 5 Refugee Support, British Red Cross, Bradbury House, Tower Street, Brunswick Business Park, Liverpool, L3 4BJ UK; 6 Department of Health Services Research, University of Liverpool, Block B Waterhouse Building, 1-5 Brownlow Street, Liverpool, L69 3GL UK; 7 Person Shaped Support, Eleanor Rathbone House, Connect Business Village, 24 Derby Road, Liverpool, L5 9PR UK; 8 Department of Public Health and Policy, Whelan Building, Brownlow Hill, Liverpool, L69 3GB UK; 9 Asylum Link Merseyside, St Anne’s Centre, 7 Overbury Street, Liverpool, L7 3HJ UK; 10 Liverpool Clinical Trials Centre, University of Liverpool, Institute of Child Health, Alder Hey Children’s NHS Foundation Trust, Liverpool, L12 2AP UK; 11 Centre for Health Economics and Medicines Evaluation, Ardudwy Hall, Bangor University, Bangor, Gwynedd LL57 2PZ UK; 12 School of Nursing and Allied Health, Liverpool John Moores University, Henry Cotton Building, 15-21 Webster Street, Liverpool, L3 2ETP UK; 13 Department of Psychological Sciences, University of Liverpool, G10 Whelan Building, Quadrangle, Brownlow Hill, Liverpool, L69 3GB UK

**Keywords:** Asylum seekers, Refugees, Mental health, Psychosocial intervention, Problem management, Lay therapists, High-income country, Pilot randomised controlled trial

## Abstract

**Background:**

Asylum seekers and refugees (AS&Rs) experience impaired mental health and wellbeing, related to stresses in their country of origin, experiences in transit and reception on arrival, including significant barriers to accessing mainstream services. Their contact with health care is often crisis-driven and mediated through non-governmental organisations (NGOs).

Problem Management Plus (PM+) is a psychosocial intervention recommended by the World Health Organisation to address distress experienced by adults affected by humanitarian crises. We are investigating its application for the first time in a high-income country.

**Methods:**

In a pilot randomised controlled trial (RCT), PM+ will be delivered to AS&Rs in contact with NGOs in Liverpool City Region, UK by lay therapists who have lived experience of forced migration. Following systematic review and stakeholder engagement, PM+ has been adapted to the local context, and lay therapists have been trained in its delivery.

We will assess the feasibility of conducting a three-arm RCT of five 90-min sessions of PM+, delivered individually or in groups by lay therapists to AS&Rs experiencing emotional distress and functional impairment, compared with each other and with usual support offered by local NGOs. Distress and impairment at baseline will be measured by the Hospital Anxiety and Depression Scale (HADS) and the WHO Disability Assessment Schedule (WHO-DAS). We aim to recruit 105 participants, 35 per arm.

Primary health outcomes are anxiety and depressive symptoms at 3 months, measured by HADS. Secondary outcomes include subjective wellbeing, functional status, progress on identified problems, presence of post-traumatic stress disorder and depressive disorder and service usage. Longer-term impact will be assessed at 6 months post baseline, on the same parameters.

We will assess the feasibility of conducting a full RCT in relation to the following elements: recruitment and retention of lay therapists and study participants; fidelity of delivery of PM+; and suitability of the study measures, including any linguistic or cultural barriers.

**Discussion:**

We will use these findings to specify the parameters for a full RCT to test the effectiveness and cost-effectiveness of PM+ in reducing emotional distress and health inequalities, and improving functional ability and wellbeing, amongst asylum seekers and refugees.

**Trial registration:**

ISRCTN, ID: ISRCTN15214107. Registered on 10 September 2019.

## Background

### Introduction

The United Nations Refugee Agency estimates that 71 million people throughout the world have been forced to flee their homes as the number of protracted conflicts has increased. This has created more than 26 million refugees worldwide, of whom an estimated 126,720 live in the UK [1]. UK Home Office figures indicate there were 34,354 asylum applications in the UK (main applicants only) in the year ending September 2019. During that year the UK offered asylum, humanitarian protection, alternative forms of leave and resettlement to 19,480 people; there were 35,043 cases pending initial decision, of which 57% were more than 6 months old [2]. Many applications are initially refused as a result of a complex system that makes it difficult for asylum seekers and refugees (AS&Rs) to provide the evidence needed to meet the criteria for gaining asylum.

AS&Rs have a higher prevalence of psychological morbidity, including depression, anxiety and post-traumatic stress disorder (PTSD), and functional impairment than other migrant groups and local majority populations [3–5]. Mental health problems are particularly prevalent amongst war refugees [6], with rates of PTSD up to 10 times higher than in the general population [7, 8]. Persistence of mental health problems after arrival in a host country is related to poor socio-economic conditions, acculturation-related stressors, economic uncertainty and ethno-racial discrimination [5, 9]. As a result, AS&Rs encounter extensive barriers to accessing health care [5, 10] and have substantial unmet mental health needs [11]. In the UK, the situation is especially problematic for asylum seekers without leave to remain who are at risk of destitution yet are required to pay for specialist health care [12, 13].

Psychosocial interventions for AS&Rs resettled in high-income countries (HICs) may provide significant benefits; however, there are few studies of good quality [14, 15]. Evidence for the applicability of psychological interventions by non-specialists in low and middle-income countries (LMICs) has increased significantly [16–19]. Many countries, including the UK, are seeking to improve health care delivery by extending the roles of health professionals [20], increasing workforce capacity and enhancing quality of care [21]. Innovations developed in LMICs, including task-sharing [22] and the Common Elements Treatment Approach [23], have the potential to address current challenges for mental health care in HICs [24], notably the lack of human resources to deliver mental health services to those in need.

Problem Management Plus (PM+), is a manualised, brief, multi-component intervention [25], recommended by the World Health Organisation (WHO) as part of its mhGAP guidelines (http://www.who.int/mental_health/mhgap/en/). It is specifically developed to be amenable to cultural and linguistic adaptation for the local context. Based on evidence-based problem-solving and behavioural techniques, the intervention is trans-diagnostic by which we mean that it applies the same intervention strategies across various common mental health problems that clients may be experiencing. Addressing multiple problems at one time through shared emotional mechanisms is efficient, reducing the practical challenge of making differential diagnoses and learning multiple treatment manuals for different mental health diagnoses [26, 27].

### Rationale

PM+ has shown significant benefit in trials in LMICs [25, 28, 29]. However, to date, there is no evidence of feasibility, effectiveness or cost-effectiveness of interventions such as PM+ offered by lay therapists to AS&Rs in HICs.

The rationale for undertaking a pilot trial of PM+ for AS&Rs, rather than proceeding to a full multi-centre trial, is that there are several areas of uncertainty regarding trial viability. These include the feasibility of recruiting and retaining AS&Rs as study participants, the fidelity of intervention delivery, and the acceptability and utility of the proposed study measures [30]. There may also be inequalities in mental health and wellbeing between AS&R groups, depending on their age, gender, nationality, education, occupational status, length of stay, access to resources and their current legal status in the UK which could inform the design of a full trial. As Northwest England has the largest number of asylum seekers in dispersal accommodation in England (9521 in September 2019) it is a suitable setting for the pilot trial.

### Preparatory work

The PROSPER Pilot trial (hereafter referred to as the PROSPER Pilot) builds on a preparatory phase aimed at developing the research team’s understanding of relevant issues, engaging with stakeholders, adapting the intervention and training the facilitators.

We conducted a systematic review (PROSPERO 2018 CRD42018104453) of barriers and facilitators to the uptake of psychosocial interventions delivered by lay therapists to improve mental health and wellbeing of asylum seekers and migrants. We also undertook six focus groups with local service providers and potential service users, and held two open meetings for stakeholders, to gather views about the mental health needs of AS&Rs and the potential utility of PM+.

As a result, we made the following contextual modifications to promote uptake and relevance of the PROSPER Pilot:

- Focus on English, Arabic, Farsi and Urdu, identified as four most common languages currently spoken by AS&Rs in Liverpool City Region
- Decision to exclude new arrivals and those in temporary accommodation: on grounds of (a) high probability of dispersal and hence unavailability for intervention and/or follow-up; and (b) low probability of being registered with a general practitioner (GP) and hence being unable to access trial safeguarding procedures
- Alteration to text of PM+ manuals to reflect life in western urban settings, rather than South Asian rural settings: e.g. ‘home’ not ‘hut’, ‘reading’ not ‘rearing poultry’, ‘visit job centre’ not ‘speak with village elder’
- Adapting the group PM+ case studies to include men
- Matching therapists and participants on basis of gender and language, but not on basis of religion, politics or culture
- Identification of accessible ‘safe spaces’ for research interviews and delivery of PM+ sessions, including availability of child care
- Reimbursement of travel expenses for lay therapists and participants
- Supervision and support of lay therapists to include boundary issues between therapy and involvement in participants’ lives, since the shared lived-experience of the asylum process takes this study beyond the boundaries that have been apparent in other contexts

### Training

Person Shaped Support (PSS) is a health and social care charity, responsible for training in and delivery of the PROSPER intervention. PSS provides a wide array of services, including Spinning World, a specialist psychological therapy service for AS&Rs and others who have experienced human-right abuses and traumatic events.

Two Wellbeing Mentors were appointed by PSS in September 2018. The following month they and their supervisor received 5 days of intensive training from two PM+ Master Trainers (from Liverpool and Amsterdam). This focussed on the delivery of the PM+ intervention strategies in both individual and group modalities, and on skills in training and supervising lay therapists. Subsequently the Wellbeing Mentors completed practice cases to embed their skills, and receive regular monthly supervision from one of the PM+ Master Trainers which will continue throughout the study.

Fifteen people with lived experience of the asylum process were offered training lay therapists, after a recruitment procedure organised through PSS. Training began in March 2019 and included education in mental disorders, basic helping skills, delivery of intervention strategies and self-care. Lay therapists received a total of 8 days of training, and were trained to deliver either individual or group PM+. This was followed by training cases and a competency assessment. Ten lay therapists successfully completed training and were assessed as competent: six in individual PM+ (two Farsi-speaking men, one Arabic-speaking man, and three women, whose languages are Urdu, Farsi and English) and four in group PM+ (one Urdu-speaking man and three women, whose languages are Arabic, Turkish and Thai).

## Methods

### Aim and objectives

This pilot trial is part of the PROSPER feasibility study, the overall aim of which is to determine whether it is possible to conduct a randomised controlled trial (RCT) in the UK of the evidence-based PM+ psychosocial intervention, delivered by lay therapists for distressed and functionally impaired asylum seekers and refugees.

The primary objective of the PROSPER Pilot is to provide preliminary information on the potential effectiveness of group or individual PM+ versus standard care for AS&Rs, assessed using severity of combined anxiety and depressive symptoms at 13 weeks post baseline, measured using the Hospital Anxiety and Depression Scale (HADS).

The secondary objectives are to provide preliminary information on the potential effectiveness and cost-effectiveness of group or individual PM+ versus standard care for AS&Rs with regards to:

- ◦ Severity of combined anxiety and depressive symptoms at 26 weeks
- ◦ Subjective wellbeing
- ◦ Functional impairment
- ◦ Progress on problems for which an individual has sought help
- ◦ Presence of post-traumatic stress disorder
- ◦ Presence of depressive disorder
- ◦ Use of services and supports from National Health Service (NHS), social care and voluntary organisations

### Design and setting

PROSPER Pilot is designed as a three-arm pilot study, with the features of a proposed future definitive RCT. Participants will be randomised to receive individual PM+, group PM+ or the control (no PM+), in a ratio of 1:1:1.

The pilot trial is being conducted in Liverpool City Region. It utilises collaborative working between three universities (University of Liverpool, Liverpool John Moores University and Bangor University) and three non-governmental organisations (NGOs) offering advice and support to AS&Rs: PSS, Asylum Link, and British Red Cross, local NGOs whose primary function is to provide advice and support for AS&Rs.

### Participants

Trial participants will be asylum seekers and refugees. This includes those with pre-asylum status; those who have been offered either discretionary or indefinite leave to remain in the UK; those whose applications for leave to remain are pending or have been refused; those with humanitarian protection; those with refugee status; stateless people; and people on the vulnerable person resettlement programme.

The other inclusion criteria are:

- Aged ≥ 18 years (self-reported)
- A score of ≥ 8 on either the depression or anxiety subscale of the HADS [31], and a score of ≥ 17 on the World Health Organisation Disability Assessment Schedule 2.0 (WHODAS) [32]
- Have conversational English, as self-assessed by the potential participant
- Being registered with a GP in Liverpool City Region
- Are willing to provide relevant socio-economic data
- Have provided written informed consent

The exclusion criteria are:

- New arrivals to the UK (less than 28 days), due to high likelihood of dispersal outside the region
- In reception centres, usually known as Initial Accommodation, and receiving temporary financial support under Section 98 of the Immigration and Asylum Act 1999 for less than 28 days, also due to high likelihood of dispersal outside the region
- Imminent risk of suicide: assessed by researchers using formal protocols with supervision and arbitration from qualified health care professionals
- Complex mental disorder (bipolar disorder/manic depression, or schizophrenia): assessed by a researcher on the basis of: participant self-reporting a diagnosis; and/or participant currently in receipt of antipsychotic medication, defined as medication listed in *British National Formulary Chapter 2, section 2.3* (bipolar disorder and mania) and *section 2.6* (psychoses and schizophrenia). If required, further clinical assessment will occur using standard formal protocols
- Cognitive impairment (moderate/severe intellectual disability, any dementia). Assessed by a researcher on the basis of participant or carer self-report
- Substance misuse: assessed by a researcher on the basis of participant response to the question: ‘are you currently having problems with alcohol, cocaine, marijuana or any other drugs?’ If the response is ‘yes’ or equivocal, then the participant will be excluded. If required, further clinical assessment will occur using standard formal protocols
- Currently receiving a formal psychological therapy, to avoid potential confounding effects

### Outcome measures

Specific outcome measures, which are candidates for inclusion in any future definitive trial of PM+ for AS&Rs, will be tested as part of PROSPER Pilot. These are summarised in Table 1.

**Table 1:** PROSPER outcome measures

Objective	Outcome measures	Time point(s) of evaluation
**Efficacy:**		
**Severity of combined anxiety and depressive symptoms**	**Hospital Anxiety and Depression Scale (HADS)** [31]	Baseline, **13-week** and 26 week follow up assessments
Functional impairment	WHO Disability Assessment Schedule (WHODAS) [32]	
Subjective wellbeing	WHO-5 Wellbeing Index [33]	
Progress with problems for which participant has sought help	Psychological Outcomes Profile (PSYCHLOPS) [34]	
Post-traumatic stress disorder (PTSD)	Post-traumatic Stress Disorder Checklist for DSM-5 (PCL-5) [35]	
Depressive disorder	9-item Patient Health Questionnaire (PHQ-9) [36]	
**Health economics:**		
Use of services and supports from NHS, social care and voluntary sectors	Adapted Client Service Receipt Inventory (CSRI) [37]	Baseline, 13-week and 26-week follow-up assessments

- HADS is a well-established, 14-item scale consisting of 2 subscales: HADS-A (anxiety; 7 items; possible score range, 0–21) and HADS-D (depression; 7 items; possible score range, 0–21). Higher scores indicate more anxiety and/or depression. HADS has been widely used across cultures; it is sensitive to change over time and has good internal consistency, reliability and validity [38]
- WHO-5 is validated in international studies for both clinical and psychometric properties and is available in many languages
- WHODAS is applicable across all health states including mental disorders. It has good validity in terms of internal consistency, test-retest reliability, and agreement with other measures of disability across countries
- PSYCHLOPS has internal consistency, convergent validity with measures of emotional distress, and is sensitive to change. It covers 3 domains: problems (2 questions), functioning (1 question) and well-being (1 question)
- PCL-5 has good psychometric properties for diagnostic accuracy and internal consistency
- PHQ-9 is based on DSM-IV depression diagnostic criteria. Total severity score ranges from 0 to 27, with 10 as conventional cut-off to diagnose depressive disorder
- The CSRI has been adapted for the PROSPER trial to include health, social care and voluntary services with the potential to be used by asylum seekers and refugees

Other elements of PROSPER Pilot will be assessed and used to inform the feasibility of conducting a full trial, as specified in Table 2.

**Table 2:** PROSPER feasibility measures

Objective	Outcome measure	Time point(s) of evaluation
To assess the feasibility of the proposed procedures for recruiting distressed AS&Rs as study participants	Number of asylum seekers and refugees (AS&Rs) recruited	Baseline
To assess feasibility of randomisation	Successful randomisation of participants	Baseline (randomisation)
To assess the feasibility of retaining study participants through to trial completion	Number of study participants in the trial (assessed in individual arms)	26 weeks
To assess the acceptability and utility of specified primary and secondary outcome measures	Completion of study measures and estimation of between group differences Evaluation of outcome measures	Baseline, 13 weeks, 26 weeks

The feasibility of progression to a definitive, multi-centre RCT will be informed by the extent to which the criteria below have been met using a go, amend, stop system, as specified in Table 3.

**Table 3:** PROSPER progression criteria

Progression criteria	Go	Amend	Stop
Recruitment of trial participants	≥ 70% of target	50–69% of target	< 50% of target
Retention of trial participants	≥ 70% retained	50–69% retained	< 50% retained
Protocol adherence	≥ 70% of intervention delivered per protocol	50–69% of intervention delivered per protocol	< 50% of intervention delivered per protocol
Completion of outcome measures	≥ 70% of measures are complete	50–69% of measures are complete	< 50% of outcome measures are complete

If the criteria meet ‘amend’ targets, reasons for this will be investigated with an aim to identify aspects that are amenable to change. If the criteria meet ‘stop’ targets, reasons will be analysed and discussed within the Project Management Group (PMG) and with independent oversight committees. If it is determined that these rates cannot be improved then a full trial will not be recommended.

Other progression criteria involving data from PROSPER Pilot that will be assessed by the research team are:

- Recruitment of supervisors and lay therapists
- Retention of lay therapists
- Acceptability of outcome measures
- Whether clinically important improvement in outcomes are plausible

### Intervention

The PM+ intervention consists of five weekly, face-to-face sessions, delivered either one-to-one or in groups. The first session opens with psychoeducation, including information on common reactions to adversity, the rationale for PM+, goal-setting, and brief motivational interviewing. Sessions 1 to 4 each introduce an intervention strategy: (1) Managing Stress (slow-breathing exercise); (2) Managing Problems (using problem solving techniques); (3) Get Going, Keep Doing (applying behavioural activation techniques) and (4) Strengthening Social Support. These strategies are applied by participants during the intervention session to problems they are facing. Each strategy is reviewed in subsequent sessions, with application of strategies between sessions encouraged to enhance learning through repetition. The final session involves a revision of learning, education on preventing relapse, and (for group PM+) ends with a culturally appropriate closing ceremony.

To enhance accessibility for groups, the group PM+ intervention is structured around locally relevant and appropriate pictorial materials and adopts a narrative format to support engagement and individual disclosure of personal difficulties which can be more difficult in a group format. Specifically, a case example of a woman or a man (depending on the gender of group participants) experiencing common functioning and emotional problems is shared each week, with participants following their progress through the PM+ group.

All PM+ sessions will take place at mutually convenient and safe locations, where support is available if required. Sessions will be delivered within organisations which have on-site staff with experience and training in managing emotional distress. No face-to-face sessions will take place in the home of either a participant or a lay therapist. There will be no special criteria for discontinuing or modifying the allocated interventions.

### Protocol adherence

Consistent with an apprenticeship model [39], protocol adherence is ensured through regular (at least fortnightly) supervision of the lay therapists provided by two Wellbeing Mentors. Involving all individual or group lay therapists in a group, supervision will last up to 3 h and will entail reviewing the progress of intervention delivery, including case management of participants and additional refresher training on intervention components. The group PM+ lay therapists will receive the same as individual PM+ lay therapists, in addition to refresher training on group facilitation skills, through role-play.

The Wellbeing Mentors are in turn provided with supervision by one of the Master Trainers, conducted at least monthly during the trial and lasting 2 h. In addition, Wellbeing Mentors will have the day-to-day support of their line manager at PSS who also participated in the 5-day PM+ training with the Master Trainers, and who participates in the monthly supervision sessions with the Master Trainer to ensure supervision consistency.

Intervention fidelity will be monitored through independent observations of 15% of randomly selected sessions of each lay therapist against tailored checklists, conducted by the Wellbeing Mentors. Session logs (per participant) will be completed by lay therapists after each PM+ session and will capture information regarding timing, length and content of sessions. The logs will be passed to the Wellbeing Mentors at weekly supervision meetings. A small number of sessions may be audio- or video-recorded as an additional assessment of intervention fidelity. Feedback from intervention observations will be used in subsequent supervision sessions to improve adherence to the intervention protocols.

Intervention compliance by trial participants will be measured by assessing adherence to the PM+ protocol with regards to attendance at sessions.

### Control arm

Participants randomised to the control arm will not be offered any PM+ but will be able to access all usual care and support offered by the participating NGOs. To control for the weekly contact that the active arms will receive, participants randomised to the control arm will be invited by the interviewing researcher to attend a local AS&R NGO of their choice. They will be put in contact with other AS&Rs from similar backgrounds and encouraged to meet together on a weekly basis for 5 weeks.

### Participant identification

Potential participants will be identified primarily through NGOs and primary care teams, all designated as Participant Information Agencies (PIAs). PIAs will be provided with a short summary of the study including the main inclusion and exclusion criteria. They will be asked to display posters and leaflets and discuss the study opportunistically with AS&Rs who access the services. All participant-facing documentation will have the necessary approvals from a Research Ethics Committee (REC).

Potential participants will be made known to the research team via one of the following methods:

- By them contacting the research team directly via telephone or email
- By agreeing to their details being given to the research team (via a participant recommendation form, completed by the PIA with the AS&R, and returned to the research team by the PIA)
- By attending a researcher-attended drop-in session at collaborating NGOs on a specific date/time, advertised by posters/leaflets/verbally

Following identification of a potential participant, a postdoctoral researcher based in the University of Liverpool, who is trained in the PROSPER trial techniques and in discussion about informed consent, will arrange a meeting to give more information about the trial.

### Informed consent

The researcher will contact the potential participant to arrange an individual face-to-face meeting. This meeting will be arranged at the convenience of the AS&R where possible and can be attended by an interpreter if required. The meeting will last between 1 and 2 h. It will take place at a convenient location which could include one of the NGO centres, a community centre, a counselling centre, NHS premises and the University of Liverpool.

Objectives, risks and inconveniences of the trial and the conditions under which it is to be conducted will be provided by the researcher. All potential participants will be given the opportunity to ask any questions that may arise, will have the opportunity to discuss the study with others and be given time to consider the information prior to agreeing to participate. It will be made clear to the participant that an eligibility assessment will be conducted once consent is given and that if the participant is found to be ineligible for any reason that they will be unable to participate.

The potential participant will be asked to read and review the Participant Information Sheet (PISC), which is available in English and the study languages of Farsi, Urdu and Arabic. Upon reviewing the document, the researcher will explain the research study to the potential participant. The PISC and the discussion with the participant will emphasise that participation in the trial is voluntary and that the participant may withdraw from the trial at any time and for any reason. Participants will also be asked for permission for the research team to share relevant data with people from the Universities taking part in the research or from regulatory authorities, where relevant. This trial does not involve collecting biological specimens for storage. The researcher is aware of the sensitive nature of the research topic and will minimise any distress caused to potential participants as a result of the discussions.

If the asylum seeker or refugee decides that they would like to participate, they will then personally sign and date the informed consent document. The document will then be signed and dated by the person obtaining consent. A copy of the informed consent document will be given to the potential participant for their records. The original document will be maintained by the research team separate from any personal identifiable information collected for any participants. A further copy will be sent to the Liverpool Clinical Trial Centre (LCTC) via secure methods if the participant is eligible for full trial participation; this will be sent separately from any participant data subsequently collected. The PISC (which includes informed consent documentation) is available from the corresponding author on request.

If the potential participant requires more time to consider involvement in the trial a further meeting can be arranged at the discretion of the researcher. If the individual does not wish to take part, their reason for not providing consent will be recorded on the PROSPER Screening Log. Once consent has been given the participant may, without being subject to any resulting detriment, withdraw from the trial at any time by revoking the informed consent.

### Eligibility and baseline assessments

Once written informed consent has been obtained, the potential participant can be assessed for eligibility, as per the criteria detailed above.

Eligibility assessment will follow a staged process. The researcher will review responses at the end of each stage and, if the potential participant is found to be ineligible, they will be informed of this and there will be no requirement for completion of the next stage.

Firstly, through discussion with the potential participant, the researcher will answer socio-demographic questions. The researcher will then assess the following exclusion criteria: complex mental disorder (bipolar disorder/manic depression, or schizophrenia); cognitive impairment (moderate/severe intellectual disability, any dementia); substance misuse; currently receiving a formal psychological therapy. If the potential participant remains eligible, they will be asked to self-complete the HADS, WHODAS and PHQ-9 questionnaires within the Eligibility Questionnaire Booklet. These questionnaires are all available in English, Arabic, Farsi and Urdu. The researcher will review the completed PHQ-9 questionnaire to assess whether the potential participant is at imminent risk of suicide. If there are any concerns regarding suicide risk, the researcher will follow the procedure outlined in the Suicidal Ideation Guidance Document.

If the potential participant is eligible following this process, the researcher will conduct the baseline assessments outlined in the following section. This will allow consistency for outcome measurement completion, and also reduce the need for attendance at additional meetings. If the researcher has any concerns or uncertainties from the non-clinical eligibility assessment above, they will contact the chief investigator (CI) or nominated deputy to discuss the case.

AS&Rs who are assessed as ineligible can be reconsidered for participation at a later date if circumstances change, e.g. if they are able to register with a GP. If this is more than 2 weeks after consent was obtained, the consent process will be repeated.

Following the completion of the eligibility assessment, the researcher will ask the eligible participant to self-complete the Baseline Questionnaire Booklet, which incorporates the remaining baseline assessments: the WHO-5, PSYCHLOPS and PCL-5 questionnaires. The CSRI Form, which has been adapted for PROSPER, will be completed by the researcher through discussion with the participant.

For a potential participant who completes the eligibility assessment process and is deemed eligible to participate in PROSPER Pilot, but where there was concern or uncertainty that necessitated the researcher contacting the CI or nominated deputy, the CI or nominated deputy will review the information provided by the participant to verify eligibility for trial participation and complete the Eligibility and Baseline CRF before randomisation occurs.

### Randomisation

Participants will be randomised using a secure, web-based randomisation programme. Randomisation lists will be generated in a 1:1:1 ratio, to individual PM+, group PM+ and control, using block randomisation with random variable block sizes.

The randomisation list will be generated by a statistician independent to the PROSPER trial. Given the open nature of the trial, it will not be possible to blind researchers, trial participants, care providers, outcome assessors or data analysts to the intervention arm to which participants are assigned.

The researcher will update the PROSPER Screening Log when a participant has been randomised. The researcher will be responsible for notifying the participant of their allocation. In the event that a participant is randomised to individual or group PM+, the researcher will inform the PSS Lead. Intervention delivery will be coordinated by PSS in collaboration with the participant and their lay therapist. The research team will notify the participant’s GP by letter of their enrolment into the trial and to what treatment arm they have been allocated.

### Assessments and follow-up

All assessments and follow-up will be conducted in line with the schedule of assessments summarised in Table 4.

**Table 4:** Schedule of assessments

	Screening and baseline	Randomisation	13-week follow-up	26-week follow-up
**Time point (weeks)**	0	0	13 ± 2	26 ± 2
**Procedures:**				
Consent, eligibility screening and confirmation				
Written and Informed consent	X			
Assess eligibility	X			
Confirm eligibility	X			
Randomisation		X		
Confirm consent		X	X	X
Data collection				
HADS	X		X	X
WHODAS	X		X	X
PHQ-9	X		X	X
PSYCHLOPS	X		X	X
PCL-5	X		X	X
WHO-5	X		X	X
CSRI	X		X	X
Adverse events				
Assessment of AEs	X		X	X

Abbreviations: *AEs* adverse events, *CSRI* Client Service Receipt Inventory*, HADS* Hospital Anxiety and Depression Scale, *PCL-5* Post-traumatic Stress Disorder Checklist for DSM-5*, PHQ-9* 9-item Patient Health Questionnaire*, PSYCHLOPS* Psychological Profiles Instrument,*, WHO-5* World Health Organisation Five Wellbeing Index, *WHODAS* World Health Organisation Disability Assessment Schedule

In the case of premature discontinuation/withdrawal, there are no additional assessments for participants.

All specified outcomes will be measured at 13 (± 2) and 26 (± 2) weeks post baseline; 13 weeks will be the primary end point: this is consistent with previous trials [28]. It allows time for intervention delivery and often may correspond to the timings of Home Office decisions on leave to remain for asylum seekers.

#### Follow-up visit: 1–13-week follow-up

This is expected to be a face-to-face appointment at 13 weeks ± 2 weeks from baseline, and include:

- Verbal confirmation of continued consent
- The participant will complete the following questionnaires within the Follow-up Questionnaire Booklet: HADS, WHODAS, PHQ-9, WHO-5, PSYCHLOPS, PCL-5
- If suicidal ideation is disclosed or suspected, the researcher will follow the steps outlined in the Suicidal Ideation Guidance document
- Recording of any adverse event information
- Researcher-led completion of the adapted CSRI
- Completion of Follow-up CRF

#### Follow-up visit: 2–26-week follow-up

This is expected to be a face-to-face appointment at 26 weeks ± 2 weeks from baseline, and to follow the same process as the follow-up appointment at 13 weeks.

All follow-up appointments will be coordinated and conducted by the trained researcher. They will conduct a preliminary review of the data collected to screen for missing data or any responses that may need further follow-up or clinical discussion. Follow-up appointments are expected to take around 1 h which should allow for completion of all data collection and review of any adverse events. If a face-to-face appointment cannot be arranged during the follow-up window then the visit can be conducted by telephone if possible. If the research team is unable to make arrangements to administer the assessments, the option of the participant self-completing the assessments and returning them by post will be explored. It is expected that participant responses will be completed during the appropriate visit window.

**Fig. 1:**
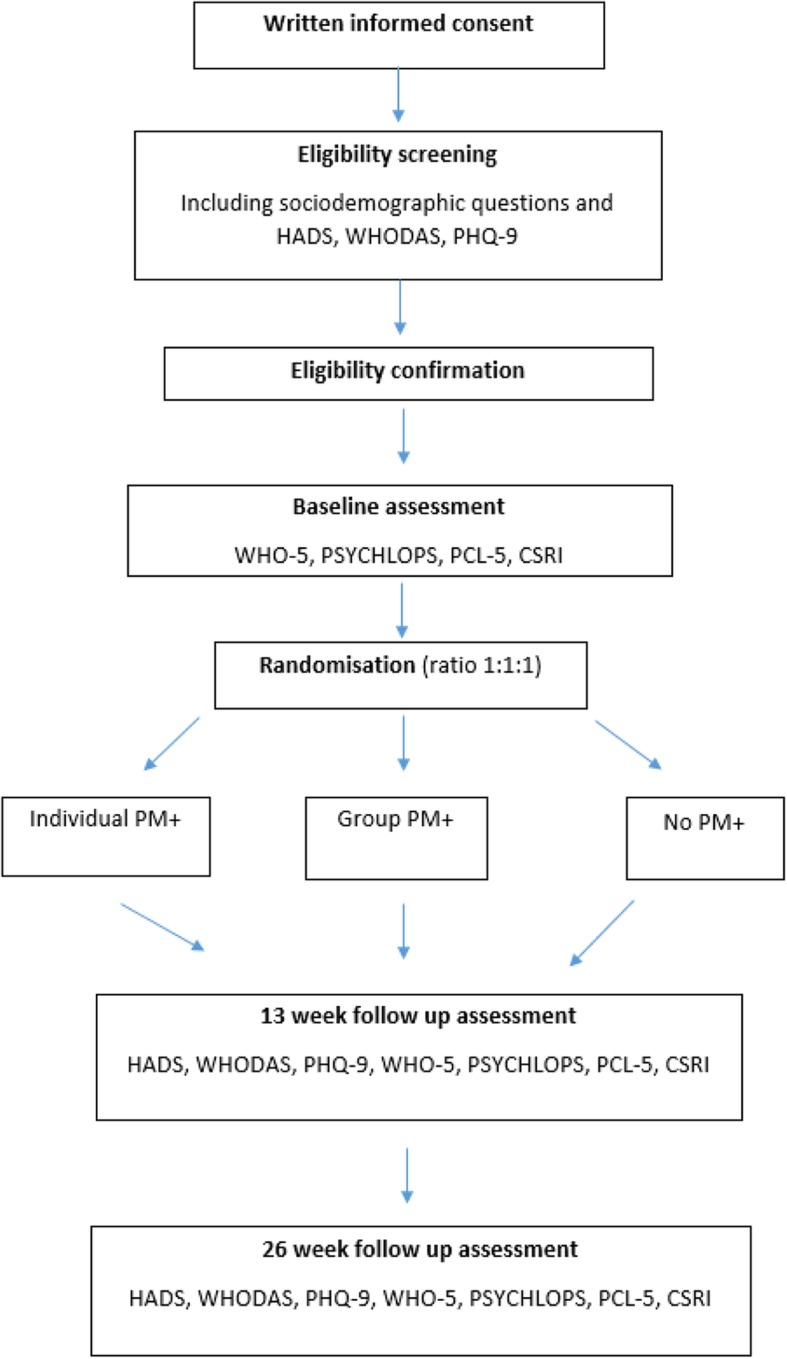
Schematic of the study design

The PROSPER Pilot study design is summarised in Fig. 1.

### Statistical considerations

A detailed statistical analysis plan (SAP) will be developed prior to the first comparative monitoring report to be presented to the Independent Data and Safety Monitoring Committee (IDSMC). The main features of these planned statistical analyses, which refer specifically to the PROSPER Pilot, are detailed below.

The aim is to recruit 105 participants, 35 to each of three arms – individual PM+, group PM+ and control. Individual sessions will be offered as gender- and language-specific, i.e. the lay therapist and the study participant will be the same gender and will be comfortable in a common language. At least four groups will be offered for the group intervention, each with up to eight or nine participants, each gender-specific, i.e. participants will all be the same gender and at least one of the lay therapists will be of the same gender as the participants.

The sample size needs to be sufficient to estimate retention levels in a definitive trial. With an expectation of 80% retention, samples of 35 participants for each of the individual, group and control arm will provide an accurate estimate of retention ± 13% (67 to 93%).

Retention rates will be assessed in each arm separately, as there may be systematic differences between them; for example, those randomised to the control arm may be less likely to remain engaged than those randomised to the individual or group arms, while those randomised to the group arm may be demotivated if faced with a lengthy wait for their group to begin.

No formal interim analysis is planned as this is a pilot study and there are no anticipated problems that are detrimental to the participant. There will be monitoring by the IDMSC, who will provide a recommendation to the Trial Steering Committee (TSC) on the continuation of the trial.

Analysis will be by the intention-to-treat principle as far as is practically possible. All analyses will be descriptive, focussed on assessing the criteria for deciding whether to progress to a full trial. All estimates of proportions will be presented with 95% confidence intervals. Rates of recruitment and attrition will be presented both for lay therapists and trial participants, along with the proportion of PM+ interventions which are successfully delivered per protocol. The proportion of missing data in the proposed trial outcome measures will be assessed.

Preliminary exploration of estimates of efficacy will involve a group-wise comparison of the primary outcome: severity of combined anxiety and depressive symptoms at 13 weeks post baseline measured using the HADS.

Socio-demographic data, and use of services and supports will be captured by the adapted Client Service Receipt Inventory [37]. This data can be used for a wide range of applications, including estimating the costs of service receipt and societal costs.

No formal testing of intervention effect will be carried out, but estimates of between-group differences between the test groups and the control in outcome measures will be presented, with 95% confidence intervals, to assess whether a clinically important improvement in outcome would be plausible in a full trial. The effect of clustering by intervention provider on outcomes in the two PM+ groups will be investigated, to inform design of a full trial with a partially nested design.

### Process evaluation and feasibility assessment

Relevance and acceptability of proposed outcomes will be tested, with a view to their incorporation or refinement for a definitive trial. These will include:

- Effectiveness of PM+, based on the primary outcome of combined HADS scores
- Cost-effectiveness of PM+ from a NHS perspective, based on the primary outcome of combined HADS scores [40, 41]
- Cost benefit from a societal perspective, given that costs and potential benefits will extend beyond the NHS to local government and voluntary sectors [42–44]
- Impact on health inequalities using the NIHR CLAHRC NWC Health Inequalities Assessment Toolkit (www.hiat.org.uk): first, within AS&R communities in relation to age, gender, nationality, education, prior occupation and asylum status; and second, between AS&Rs and national populations, comparing mental health status (anxiety, depression PTSD and wellbeing) with UK population norms, with reference to published psychiatric morbidity data [45]

The feasibility of the 13- and 26-week time points will be assessed, with specific reference to rates of participant attrition.

Researchers will undertake a systems-based *process evaluation* [46], beginning 3 months into the PROSPER Pilot, to: understand service provider and participant experiences and perspectives on acceptability, efficiency, implementation and development of PM+; understand service-users’ perceptions and experiences of accessing and participating in PM+; explore how PM+ fits into existing health/social care systems; and understand change-process dynamics including barriers and facilitators to implementing PM+. An ethnographic method will be adopted including observation of PM+ implementation alongside semi-structured interviews and focus group discussions with key stakeholder groups such as lay therapists, Wellbeing Mentors, PM+ participants, representatives from NGOs working with AS&R communities, health professionals and commissioners from Liverpool City Region. Heterogeneity within the population will be considered and whether the intervention’s feasibility and effectiveness may differ by demography or asylum status, and how this may influence the choice of target population for our proposed definitive trial.

Analysis will be based on narrative synthesis, combining data tabulation and narrative techniques. This will involve iterative review and refinement in order to reach agreement on a set of general propositions in relation to the data. The perspectives of Normalisation Process Theory [47, 48] will be used to assess the potential for implementing a full RCT, focussing on the progression criteria set out above.

### Discontinuation and withdrawal

In consenting to the trial, participants agree to all trial activities including administration of trial intervention and follow-up assessments/visits and data collection. Every effort will be made to facilitate the completion of these for every recruited participant. If it is not possible to complete these activities (or it is deemed inappropriate) the reasons why should be documented.

Participants may discontinue the study intervention for reasons including, but not limited to:

- Participant-led, i.e. request by the participant
- Researcher/clinician/lay therapist-led:
  - Any change in the participant’s condition that justifies the discontinuation of the intervention in the researcher/clinician/lay therapist’s opinion
  - Reasons of non-adherence or non-compliance with study intervention or other trial procedures, e.g. unable to complete course of PM+
  - Participant meets an exclusion criterion (either newly developed or not previously recognised)

Discontinuation from PM+ does not mean discontinuation of the study altogether, and the remaining study procedures, i.e. 13- and 26-week follow-up visits and data collection, and process evaluation, will be completed as indicated in the protocol (unless consent is specifically withdrawn).

Participants are free to withdraw from follow-up at any time without providing a reason, though a reason should be recorded if one is given. Those who wish to withdraw from further follow-up will have the data collected up to the point of that withdrawal included in the analyses. The participant will not contribute further data to the study and the LCTC will be informed, via email to the LCTC and via completion of a Withdrawal CRF to be returned to the LCTC within 24 h. Death of a participant would be recorded on a Withdrawal CRF and a Death CRF.

For participants moving from the area, every effort will be made for the participant to be followed up and to complete their remaining study appointment(s) remotely.

A participant will be considered lost to follow-up if they fail to return for any scheduled visits and are not contactable by the site research team. If a participant fails to attend/facilitate a required study visit the following actions must be taken:

- The researcher will attempt to contact the participant and reschedule the missed visit (be conscious of acceptable windows for collecting valid data) and advise the participant on the importance of maintaining the assigned visit schedule
- Before a participant is deemed to be lost to follow-up, the research team will make reasonable effort to regain contact with the participant
- If the participant continues to be unreachable they should be considered withdrawn from the study with a primary reason of lost to follow-up and this should be recorded on the Withdrawal CRF

### Confidentiality and access to data

Forms which contain participant identifiers will be stored separately from the Case Report Forms (CRFs). The database will be secured with password protection. Participants’ data will not be released outside of the study without the written permission of the participant, documented in the consent form.

The University of Liverpool and Bangor University are registered as data controllers with the Information Commissioner’s Office.

### Safety and monitoring

Safety assessments will be based on information disclosed by the participant throughout trial duration and by those who have knowledge of their welfare, including GPs, other health professional and NGO members. The CI and other research staff are responsible for monitoring and reporting all adverse events.

Ancillary and post-trial care will be the responsibility of the participant’s registered GP.

Data will be centrally monitored by the LCTC to promote data quality. Monitoring processes are documented in the ‘Trial Monitoring Plan’ and can be made available from the authors on request. If necessary, on-site monitoring visits can be triggered and will be carried out by either the LCTC or the sponsor representative.

Safety information and data will be independently monitored by the IDSMC. The IDSMC is chaired by an independent senior clinical academic, and includes an independent methodological expert and an experienced service user. The IDSMC will report to the TSC, and hence to the NIHR Public Health Research Programme Board. The composition and terms of reference for both the IDSMC and the TSC are available from the authors on request.

### End of trial

The end of the trial is defined to be the date on which data for all participants is locked and data-entry privileges are withdrawn from the trial database. However, the trial may be closed prematurely by the TSC on the recommendation of the IDSMC.

### Dissemination

Using established procedures for knowledge exchange we will disseminate the findings of our research through:

- Dedicated project web-page and social media sites
- Feedback to participants, both service users and providers
- Presentations to stakeholder groups including service users and providers, policy-makers and commissioners, funders and benefactors
- Presentations to national asylum seeker and refugee NGOs
- Presentations at clinical academic conferences
- Report for *NIHR Public Health Research* journal
- Submission of research papers to high-impact peer-reviewed journals

## Discussion

The PROSPER feasibility study and Pilot Trial should generate new knowledge of benefit to the NHS and to society in general. This study will ascertain whether lay therapists based in NGOs can be trained to deliver PM+ with demonstrable evidence of capacity. It should provide early indications as to whether PM+ can lead to improvements in mental health and function for distressed AS&Rs in current UK settings. It should identify potential new pathways for access to care for these vulnerable groups, overcoming existing barriers such as accessibility of delivery locations and language barriers.

There is currently a lack of evidence on feasibility of conducting research into psychosocial interventions in these circumstances, and this study will address this gap in the evidence base. We anticipate that the study will provide clear evidence on the key parameters needed for a definitive RCT in this field. Such a definitive trial has the potential to improve mental health, wellbeing and functional ability amongst AS&Rs, and to reduce health inequalities. This is likely to lead to more equitable and effective use of health care, with a shift from receiving emergency care to managed, proactive and preventive care. From a societal perspective, cost-effectiveness and cost-benefit analyses following the definitive trial will indicate the extent to which the intervention confers both direct and indirect benefits. Public and patient involvement should ensure that the project delivers high-quality, original evidence that has the potential to have a significant impact on the design of the definitive intervention and, subsequently, on policy and practice.

From an international perspective, our findings should have relevance for other HICs hosting refugees, as well as for WHO recommendations on the use of PM+ with AS&R communities who experience significant adversities.

## Trial status

- Protocol version V5.0, dated: 11 December 2019
- Recruitment start: 27 November 2019
- Recruitment completion (expected): 31 May 2020

## Data Availability

Fully anonymised, participant-level datasets and statistical codes can be made available upon reasonable request once the final results of the trial have been published.

## Abbreviations

AE: Adverse event
AS&R: Asylum seeker and refugee
CI: Chief investigator
CRF: Case Report Form
CSRI: Client Service Receipt Inventory
DSM-5: *Diagnostic and Statistical Manual of Mental Disorders (5th edition)*
GP: General practitioner
HADS: Hospital Anxiety and Depression Scale
HICs: High-income countries
HRA: Health Research Authority
IAPT: Increasing Access to Psychological Therapies
IDSMC: Independent Data and Safety Monitoring Committee
LCTC: Liverpool Clinical Trials Centre
LMICs: Low- and middle-income countries
NGO: Non-governmental organisation
NHS: National Health Service
NRES: National Research Ethics Service
NIHR CRN: National Institute for Health Research Clinical Research Network
PHQ-9: 9-item Patient Health Questionnaire
PIA: Participant Information Agency
PM+: Problem Management Plus
PMG: Project Management Group
PSS: Person Shaped Support
PSYCHLOPS: Psychological Profiles Instrument
PTSD: Post-traumatic stress disorder
RCT: Randomised controlled trial
REC: Research Ethics Committee
SAE: Serious adverse event
TSC: Trial Steering Committee
WHO: World Health Organisation
WHO-5: World Health Organisation Five Wellbeing Index
WHODAS: World Health Organisation Disability Assessment Schedule

## References

[1] United Nations Refugee Agency (2018). Global Trends: Forced Displacement.

[2] Home Office. How many people do we grant asylum or protection to? https://www.gov.uk/government/publications/immigration-statistics-year-ending-september-2019/how-many-people-do-we-grant-asylum-or-protection-to. Accessed 9 Dec 2019.

[3] Lindert J, Ehrenstein OS, Priebe S, Mielck A, Brähler E (2009). Depression and anxiety in labor migrants and refugees: a systematic review and meta-analysis. Soc Sci Med.

[4] Close C, Kouvonen A, Bosqui T, Patel K, O'Reilly D, Donnelly M (2016). The mental health and wellbeing of first generation migrants: a systematic-narrative review of reviews. Glob Health.

[5] Priebe S, Giacco D, El-Nagib R (2016). Public health aspects of mental health among migrants and refugees: a review of the evidence on mental health care for refugees, asylum seekers and irregular migrants in the WHO European Region.

[6] Bogic M, Njoku A, Priebe S (2015). Long-term mental health of war-refugees: a systematic literature review. BMC Int Health Hum Rights.

[7] Fazel M, Wheeler J, Danesh J (2005). Prevalence of serious mental disorder in 7000 refugees resettled in western countries: a systematic review. Lancet.

[8] Slewa-Younan S, Uribe Guajardo MG, Heriseanu A, Hasan T (2015). A systematic review of Post-traumatic Stress Disorder and depression amongst Iraqi refugees located in western countries. J Immigr Minor Health.

[9] George U, Thomson MS, Chaze F, Guruje S (2015). Immigrant mental health, a public health issue: looking back and moving forward. Int J Environ Res Public Health.

[10] van der Boor CF, White R (2020). Barriers to accessing and negotiating mental health services in asylum seeking and refugee populations: the application of the candidacy framework. J Immigr Minor Health.

[11] Bradby H, Humphris R, Newall D, Phillimore J (2015). Public health aspects of migrant health: a review of the evidence on health status for refugees and asylum seekers in the European Region.

[12] Kang C, Tomkow L, Farrington R (2019). Access to primary health care for asylum seekers and refugees: a qualitative study of service user experiences in the UK. Br J Gen Pract.

[13] Nellums L, Rustage K, Hargreaves S, Friedland J. Access to healthcare for people seeking and refused asylum in Great Britain. (Equality and Human Rights Commission Report 121), 2019. https://www.equalityhumanrights.com/sites/default/files/research-report-121-people-seeking-asylum-access-to-healthcare-evidence-review.pdf. Accessed 10 Dec 2019.

[14] Nosè M, Ballette F, Bighelli I, Turrini G, Purgato M, Tol W (2017). Psychosocial interventions for post-traumatic stress disorder in refugees and asylum seekers resettled in high-income countries: systematic review and meta-analysis. PLoS One.

[15] Netherlands Ministry of Foreign Affairs. Mind the Mind Now – International Conference on Mental Health & Psychosocial Support in Crisis Situations: Background Document and Recommendations. https://www.government.nl/ministries/ministry-of-foreign-affairs/documents/publications/2019/09/30/international-conference-on-mhpss-in-crisis-situations-background-document-and-recommendations . Accessed 6 Nov 2019.

[16] Wiley-Exley E (2007). Evaluations of community health care in low and middle income countries: a 10 year review of the literature. Soc Sci Med.

[17] Rahman A, Malik A, Sikander S, Roberts C, Creed F (2008). Cognitive behaviour therapy-based intervention by community health workers for mothers with depression and their infants in rural Pakistan: a cluster-randomised trial. Lancet.

[18] Bolton P, Lee C, Haroz E, Murray L, Dorsey S, Robinson C (2014). A transdiagnostic community based mental health treatment for comorbid disorders: development and outcomes of a randomized controlled trial among Burmese refugees in Thailand. PLoS Med.

[19] Turrini G, Purgato M, Acarturk C, Anttila M, Au T, Ballette F (2019). Efficacy and acceptability of psychosocial interventions in asylum seekers and refugees: systematic review and meta-analysis. Epidemiol Psychiatr Sci.

[20] Delamaire M, Lafortune G. Nurses in Advances Roles: a description and evaluation of experiences in 12 developed countries. OECD Working Papers, 2010: http://www.oecd-ilibrary.org/social-issues-migration-health/nurses-in-advanced-roles_5kmbrcfms5g7-en. Accessed 12 Dec 2019.

[21] Ham C, Berwick D, Dixon J (2016). Improving quality in the English NHS: a strategy for action.

[22] Padmanathan P, De Silva MJ (2013). The acceptability and feasibility of task-sharing for mental healthcare in low and middle income countries: a systematic review. Soc Sci Med.

[23] Murray LK, Dorsey S, Haroz E, Lee C, Alsiary MM, Haydary A (2014). A common elements treatment approach for adult mental health problems in low- and middle income countries. Cognit Behav Pract.

[24] Sashidharan SP, White R, Mezzina R, Jansen S, Gishoma D (2016). Global mental health in high-income countries. Br J Psychiatry.

[25] Dawson KS, Bryant RA, Harper M, Kuowei Tay A, Rahman A, Schafer A (2015). Problem Management Plus (PM+): a WHO transdiagnostic psychological intervention for common mental health problems. World Psychiatry.

[26] Wilamowska ZA, Thompson-Hollands J, Fairholme CP, Ellard KK, Farchione TJ, Barlow DH (2010). Conceptual background, development, and preliminary data from the unified protocol for transdiagnostic treatment of emotional disorders. Depress Anxiety.

[27] Bullis JR, Fortune MR, Farchione TJ, Barlow DH (2014). A preliminary investigation of the long-term outcome of the Unified Protocol for Transdiagnostic Treatment of Emotional Disorders. Compr Psychiatry.

[28] Rahman A, Hamdani SU, Awan NR, Bryant RA, Dawson KS, Khan MF (2016). Effect of a multicomponent behavioral intervention in adults impaired by psychological distress in a conflict-affected area of Pakistan: a randomized clinical trial. JAMA..

[29] Bryant RA, Schafer A, Dawson KS, Anjuri D, Mulili C, Ndogoni L (2017). Effectiveness of a brief behavioural intervention on psychological distress among women with a history of gender-based violence in urban Kenya: a randomised clinical trial. PLoS Med.

[30] Gabriel P, Kaczorowski J, Berry N (2017). Recruitment of refugees for health research: a qualitative study to add refugees’ perspectives. Int J Environ Res Public Health.

[31] Zigmond AS, Snaith RP (1983). The Hospital Anxiety and Depression Scale. Act Psychiatr Scand.

[32] Andrews G, Kemp A, Sunderland M, Von Korff M, Ustun TB (2009). Normative data for the 12 item WHO Disability Assessment Schedule 2.0. PLoS One.

[33] Topp CW, Østergaard SD, Søndergaard S, Bech P (2015). The WHO-5 Well-Being Index: a systematic review of the literature. Psychother Psychosom.

[34] Ashworth M, Shepherd M, Christey J, Matthews M, Wright K, Parmentier H, et al. A client-centred psychometric instrument: the development of PSYCHLOPS (Psychological Outcome Profiles). Counsel Psychother Res. 2004;4:27–33.

[35] Blevins CA, Weathers FW, Davis MT, Witte TK, Domino JL (2015). The Posttraumatic Stress Disorder Checklist for DSM-5 (PCL-5): development and initial psychometric evaluation. J Trauma Stress.

[36] Kroenke K, Spitzer RL, Williams JB (2001). The PHQ-9: validity of a brief depression severity measure. J Gen Int Med.

[37] Beecham J, Knapp M, Thornicroft G, Brewin C, Wing J (1992). Costing psychiatric services. Measuring mental health.

[38] Herrmann C (1997). International experiences with the Hospital Anxiety and Depression Scale: a review of validation data and clinical results. J Psychosom Res.

[39] Murray LK, Bolton P, Dorsey S, Jordans MJ, Rahman A, Bass J (2011). Building capacity in mental health interventions in low resource countries: an apprenticeship model for training local providers. Int J Ment Health Syst.

[40] Drummond MF, Sculpher MJ, Claxton K, Stoddart GL, Torrance GW (2015). Methods for the economic evaluation of health care programmes.

[41] National Institute for Health and Care Excellence. Methods for the development of NICE public health guidance. 3rd ed. London; 2012. https://www.nice.org.uk/process/pmg4/resources/methods-for-the-development-of-nice-public-health-guidance-third-edition-pdf-2007967445701. Accessed 6 Mar 2020. 27905711

[42] McIntosh E (2010). Applied methods of cost-benefit analysis in health care (Vol. 4).

[43] Pearce D, Atkinson G, Mourato S. Cost-benefit analysis and the environment: recent developments. Organisation for Economic Co-operation and Development. 2006.

[44] Sugden R, Williams A (1978). The principles of practical cost-benefit analysis.

[45] McManus S, Bebbington P, Jenkins R, Brugha T. Mental health and wellbeing in England. Adult Psychiatric Morbidity Survey 2014: Health and Socieal Care Information Centre; 2016. https://assets.publishing.service.gov.uk/government/uploads/system/uploads/attachment_data/file/556596/apms-2014-full-rpt.pdf. Accessed 6 Mar 2020.

[46] Moore GF, Audrey S, Barker M, Bond L, Bonell C, Hardeman W (2015). Process evaluation of complex interventions: Medical Research Council guidance. BMJ.

[47] Murray E, Treweek S, Pope C, MacFarlane A, Ballini L, Dowrick C (2010). Normalisation process theory: a framework for developing, evaluating and implementing complex interventions. BMC Med.

[48] Finch TL, Rapley T, Girling M, Mair FS, Murray E, Treweek S (2013). Improving the normalization of complex interventions: measure development based on normalization process theory (NoMAD): study protocol. Implement Sci.

